# Effects of attention on the processing of physical and abstract auditory regularities: An exploratory MMN study

**DOI:** 10.1016/j.heliyon.2024.e33182

**Published:** 2024-06-17

**Authors:** Petri Paavilainen, Marianne Ilola

**Affiliations:** Cognitive Brain Research Unit, Department of Psychology and Logopedics, University of Helsinki, Finland

**Keywords:** Mismatch negativity, (MMN), Event-related potentials, (ERPs), Attention, Memory, Auditory information processing

## Abstract

The mismatch-negativity (MMN) component of the auditory event-related potential (ERP) reflects preattentive memory mechanisms encoding various types of regularities in the auditory environment. In an oddball paradigm, two types of deviant stimuli (in separate blocks) were presented among frequent standard stimuli: physical deviants (higher in pitch than the standards) and more complex, “abstract” deviants (tone pairs descending in pitch, presented among ascending standard tone pairs). The attentional load of the participant was manipulated under three conditions, where the participants either (1) watched a silent video, (2) played a computer game (Tetris) or (3) attended to the auditory stimuli and tried to detect infrequent target stimuli which were of lower intensity than the standard stimuli. The goal was to find out, whether the possible attention effects (suppression/enhancement) on the MMN are similar or different to stimuli requiring the extraction of either physical or abstract invariances. Both the physical and abstract deviants elicited in all conditions MMNs but no statistically significant amplitude differences between the conditions were found. The N2b and P3b components were elicited only in the attend condition and only by the soft target tones. The results further confirm that the MMN is a robust response to various types of regularity violations, showing no major effects of attentional manipulations. The results also suggest that the most commonly used primary task in MMN experiments, watching a silent video, usually keeps the participants' attention well enough directed away from the auditory stimuli. However, in cases where a cognitively more demanding but still participant-friendly primary task is needed, a simple computer game such as Tetris can be used, enabling better control of the participants’ attention and vigilance.

## 1 Introduction

### 1.1 Physical and abstract-feature MMNs

Automatic, attention-independent auditory processing in the human brain can be investigated using the *mismatch-negativity* (MMN) component of the auditory event-related potential (ERP). This component has proven to be valuable both in basic research (for reviews, see Refs. [[1], [2], [3]]) and as a potential clinical tool in determining brain dysfunctions related to various neurological and psychiatric disorders [2,4]. The so-called “oddball paradigm” is typically used in MMN studies: the participant is presented with physically identical auditory stimuli (“standards”). These stimuli are occasionally replaced by, e.g., tones of a different pitch (“deviants”). A negative deflection, the MMN, can be observed at electrodes over the frontal and central scalp areas between 100 and 200 ms as a response to stimulus deviance. The principal cerebral generators of the MMN are located at the auditory cortices. Consequently, using a nose reference, a polarity-inverted MMN can be recorded at electrodes below the level of the Sylvian fissure [2].

According to the original MMN theory, developed by Risto Näätänen (e.g. Refs. [5,6]), the physical features of the standard tones (e.g., pitch, duration) are stored in short-lived sensory-memory traces at the auditory cortices. The MMN reflects a mismatch between the properties of the deviant tone and the current contents of the memory trace. MMN is elicited even when the participant is not attending to the auditory input, indicating that the processing underlying the MMN generation is more or less preattentive or automatic in character. An “ignore condition”, where the participant is concentrating on a primary task not related to the simultaneously presented auditory stimuli (e.g., he/she is watching a silent video), is typically used in MMN recordings. The ignore condition is intended to prevent MMN from being obscured by other, attention-related ERP components [7]. The functional role of the MMN mechanism was proposed to be in initiating an attention switch to sudden changes in the auditory environment. This would ensure that such potentially important events are noticed even if the attention was initially directed elsewhere. The ERP indicator of the involuntary attention switching is the P3a component, following the MMN [8,9].

However, as the MMN research advanced, it was found that the proposed memory traces encoded also more complex information than just simple physical features. The preattentive auditory analysis mechanisms could detect also certain “higher-order” or “abstract” regularities, based on the relationships between different physical stimulus features (for a review, see Ref. [10]). In the pioneer study by Saarinen et al. [11], the authors used tone pairs (two 60-ms tone pips separated by a 40-ms silent gap) as their stimuli. The tone pairs were presented with 640-ms intervals. The position of the tone pairs randomly varied along the pitch dimension, there being no repeating, physically identical standard stimulus. However, all the standard pairs had a common higher-order feature (see their Condition 1): the *direction* of the within-pair pitch change. The standard pairs were ascending (i.e., the first tone of a pair was lower in pitch than the second tone), whereas the deviant pairs were descending. Thus, the standard-stimulus invariance was based on a *relationship* between the physical features of the two tones forming a pair. The deviant pairs were found to elicit MMN. The interpretation of the result was that the preattentive stimulus representations derived also abstract features or simple concepts (“rise”, “fall”) from a stream of physically varying events.

In his influential reinterpretation of MMN, Winkler [12] proposed that the brain mechanisms underlying the generation of MMN are automatically extracting various types of rules or regularities from the auditory environment. The regularities are encoded in neural models that are updated when stimuli violating the regularities are encountered, the MMN signaling the updating process.

### 1.2 The dispute over attention effects on MMN

Although the elicitation of the MMN is currently generally considered, at least to a large extent, automatic, the attention-independence of the MMN has been an object of extensive controversy during the past decades (for a review, see Ref. [13]). In the early MMN studies, typically reading a book was used in the ignore condition as the primary task. To avoid muscular artifacts related to turning pages, the reading task was soon replaced with a video-watching task, where the participant is typically watching a silent movie with subtitles. Video watching is currently the most commonly used primary task in MMN measurements. ERP studies and MMN experiments, in particular, are typically rather long, as getting an adequate number of summations for the ERPs to the infrequent deviants takes time. Watching an interesting movie during the measurement is an easy and comfortable primary task for the participant.

However, it is not clear how well the video can all the time keep the participant's attention directed away from the auditory stimuli as watching a video does not usually produce an especially high perceptual and attentional load on the participant. Lower levels of perceptual load are known to increase the distracting effects caused by task-irrelevant stimuli or internal sources of distraction such as mind-wandering (see, e.g., Refs. [14,15]. As the video soundtrack is usually turned off to improve the audibility of the oddball tones, it may be difficult to follow the movie all the time just based on the visual input and subtitles. There may be periods when the participant's attention is wandering, and they may be more or less consciously attending to the tones. This may lead to the elicitation of attention-related ERP components such as the N2b ([16,17] and P3b [18], which may obscure the interpretation of the MMN results. In the worst case, the participant may find the movie chosen uninteresting and give up actively attending it. It is usually difficult for the experimenter to follow in real time the participant's actual behavior and cognitive processing during the video-watching task. Recently, however, Hsu et al. [19] studied the MMNs to abstract deviant tone pairs using a more controlled video-watching task. The participant's task was to count certain target events in the video (e.g., appearances of characters in an animated video) and to report their number at the end of the video. They found that the MMN to deviant tone pairs was unaffected compared to a condition where attention was directed to the tone pairs.

It has been shown, however, that the amplitude of MMN may, at least to some extent, be modulated by the direction of attention. In the 1990s, several MMN experiments used dichotic listening tasks where oddball tone series were delivered simultaneously to the participant's left and right ear (e.g., Refs. [[20], [21], [22], [23]]. The participant was instructed to attend to the tones presented, e.g., in the right ear, and to press a button to deviants in that ear. This procedure controlled better the direction of attention. In some studies, it was observed that the amplitude of the MMN to the attended-ear deviants seemed to be enhanced when compared to those presented to the non-attended ear. However, it was difficult to resolve whether this was due to the more intense activation of the MMN generator *per se* or whether it was produced by the attention-related N2b component, partially overlapping the MMN. On the other hand, some studies reported attenuation or even disappearance of MMN to the deviants presented in the non-attended ear. These effects, in turn, seemed to be somewhat different depending on the physical attribute eliciting the MMN (i.e., frequency, intensity) and which attribute was the target in the attended ear (for a review, see Ref. [24]).

Attention might, however, influence differentially MMNs to deviances in physical and abstract stimulus features. One dichotic-listening study [25] used Saarinen et al.‘s [11] abstract MMN paradigm. Standard and deviant pairs were delivered with short SOAs (stimulus-onset-asynchrony) randomly to left and right ears, their attributes (ascending/descending) being opposite in the two ears. When the left-ear pairs were attended to, MMN was elicited by the (unattended) right-ear deviant pairs. However, when attention was directed to the right ear, there was no MMN to the left-ear deviant pairs. As an explanation for this discrepancy, the authors speculated that the processing of abstract attributes might differ between the two ears, as the left and right-ear inputs are predominantly processed in the opposite hemispheres. The left hemisphere might be more specialized in this kind of analytical processing than the right hemisphere. Therefore, the extraction of abstract features in the right-ear input might be affected less by the strong focusing of attention to the opposite ear. Whether the abstract-feature MMNs might be generally more prone to attentional influences than the physical-feature MMNs, has not been systematically studied.

Several studies have compared the MMNs recorded to deviant tones during various cognitively demanding *visual* tasks (e.g., n-back tasks, visuospatial tracking tasks; for a summary and meta-analysis, see Refs. [26,27]). The MMN elicitation seems to be a rather robust phenomenon although many studies have reported some attenuation of MMN amplitude as a result of increasing difficulty of the visual task (e.g. Ref. [28]). Haroush et al. [26] concluded that the MMN mechanism is maintained even during conditions requiring very high visual attention, although they found that the MMN amplitude and latency were susceptible to momentary attentional fluctuations as reflected in success or failure to identify a visual target (see also [27]).

Although probably better in controlling the direction of attention, the dichotic-listening paradigms are rather stressful for the participants, as they require extended periods of concentrating on the rather boring tone beeps. Similarly, most types of demanding visual tasks used in the aforementioned studies can be quite exhausting, compared to the basic video-watching task. This may make their routine use in MMN studies (especially in clinical ones) difficult. One possibility to better control the direction of attention in future MMN studies when needed might be to replace video watching with some attentionally and cognitively more demanding, but still motivating primary task, such as an interesting visual computer game. A game requiring continuous performance and alertness might better keep the attention directed on the visual input and prevent it from wandering to the auditory modality.

### 1.3 The goals of the present study

The present exploratory study was designed to compare the MMNs elicited by two types of deviants in three conditions with different types of primary tasks (2 × 3 design). In the physical-MMN blocks, infrequent pitch changes were used as deviants. In the abstract-MMN blocks, we used the Saarinen et al. [11] setup where the deviant events were occasional descending tone pairs presented among ascending tone pairs. Our first goal was to find out, whether the possible attention effects (suppression/enhancement) on the MMN are similar or different to stimuli requiring the extraction of simple physical invariances or more complex, abstract invariances.

The second goal was to study the effects of three different types of primary tasks on the MMN. The tasks were video watching, a visual computer game, and an auditory detection task. Video watching was used as a kind of baseline condition, as it is the most commonly used primary task in MMN studies. In the computer game condition, we used the classical Tetris game [29] as the participant's primary task. In the auditory detection task, the participant's attention was directed to the auditory stimuli to find out, whether attention might enhance the MMN. In most detection tasks used in the previous studies, the deviants eliciting the MMN have also been the target stimuli requiring the participant's response. The targets, however, usually elicit in addition to the MMN, also the N2b component. This component is related to decision-making and response, and often partially overlaps the MMN. Consequently, it may be difficult to disentangle the possible attention effects on MMN from the contribution of N2b. To reduce the possible N2b overlap, in the present stimulus streams there were two types of deviants. The non-target deviants, whose MMN was the main object of the study, were either pitch deviants (physical MMN blocks) or descending stimulus pairs (abstract MMN blocks). The target deviants, which the participants had to detect in the detection task, were otherwise similar to the standard stimuli but of lower intensity. It was hypothesized that the N2b should be elicited mainly by the target deviants whereas its contribution should be absent or at least smaller in the ERPs to the non-target deviants, allowing the possible attention-related effects on the MMN to be seen without the N2b contamination.

As for our choice for the computer game, Tetris is a classical game, known for its high immersiveness despite the very simple task of the player: The player tries to complete lines by moving differently shaped forms, which descend onto the screen. Successful Tetris playing requires continuous attention to the visual modality, as the descending forms require immediate responses from the player. Thus, the game can on good grounds be considered as a cognitively more loading task than the more or less passive video watching. As Tetris is very easy to learn (and already familiar to many people), it was considered a good choice for the game. Moreover, as the player's gaze is directed most of the time to a rather narrow area on the screen and the game requires only the use of three fingers, it should not induce large eye or head movements or motor artifacts that could distort the ERPs.

Tetris has been previously used as a primary task at least in one MMN study, investigating the effects of sleep deprivation on MMN ([30]. However, to our knowledge, there exist no previous studies where the MMNs obtained during the game have been compared to those obtained in other kinds of primary tasks. As the MMN is increasingly used both in basic research and various clinical paradigms, it would be important to know whether video watching is valid enough to be used in the future or whether it should be replaced with some other, more controlled but participant-friendly task such as a computer game. It is also important to know whether different types of MMNs (e.g., physical vs. abstract) might be differentially influenced by the type of the primary task. For example, Grimm & Escera [31] proposed that several types of hierarchical mechanisms, operating on different neural levels, are involved in responding to various regularity-violating events, depending on the complexity of the regularities. These mechanisms might be differentially affected by attentional demands. There is also some evidence that the MMNs to more complex regularities might be more vulnerable to neurodegenerative factors related to, e.g., Alzheimer's disease and aging [32,33]. Thus, also from the point of potential clinical applications of MMN, it would be important to know whether different kinds of attentional manipulations might have different effects depending on the type of stimuli eliciting the MMN.

## 2 Materials and methods

### 2.1 Participants

15 adults (10 females, 5 males; age range 18–45 yrs, mean 25 yrs; one left-handed) participated in the experiment. All the participants reported having normal hearing. Written informed consent was obtained from all participants included in the study. They received either a 15 € reward or course credits for their participation in the study. The experimental procedure was carried out in accordance with the Declaration of Helsinki and was ethically accepted by the University of Helsinki Ethical Review Board in Humanities and Social and Behavioral Sciences (approval number: 4/2022).

### 2.2 Stimuli and procedure

Two types of stimulus blocks were delivered to the participants (see Table 1). In the *physical MMN blocks*, the stimuli were sinusoidal 100-ms tone pips (with 10-ms linear rise and fall times). Each block consisted of three types of stimuli. The tonal frequency of the *standard stimuli* (p = 0.8) was 658 Hz and they were presented at a comfortable loudness level (about 70 dB SPL). The frequency of the *pitch deviants* (p = 0.1) was 740 Hz and their intensity same as that of the standards. The *soft tones* (p = 0.1) had the same pitch as the standards, but their intensity was 8 dB lower.

**Table 1:** The parameters of the auditory stimuli in physical and abstract MMN blocks.

Physical-MMN blocks	standard tones	pitch deviants	soft tones
	658 Hz p = 0.8	740 Hz p = 0.1	658 Hz p = 0.1
			intensity 8 dB lower

Abstract-MMN blocks	standard pairs	abstract deviants	soft tone pairs
	523–587 Hz	587-523 Hz	523–587 Hz
	587–659 Hz	659-587 Hz	587–659 Hz
	659–740 Hz	740-659 Hz	659–740 Hz
	740–830 Hz	830-740 Hz	740–830 Hz
	830–932 Hz	932-830 Hz	830–932 Hz
	total p = 0.8	total p = 0.1	total p = 0.1
			intensity 8 dB lower

In the *abstract MMN blocks*, the stimuli were tone pairs consisting of two 60-ms sinusoidal tones (with 5-ms rise and fall times), separated by a 40-ms silent gap. Each block consisted of three types of stimulus pairs. The *standard pairs* were ascending in direction, i.e., the second tone of the pair was one whole note higher than the first tone. There were five exemplars of ascending pairs (p each = 0.16, total p = 0.8), located on different pitch levels (see Table 1). The *abstract deviants* (p each = 0.02, total p = 0.1) were reversals of the corresponding standard pairs, i.e., they were descending in direction. The *soft tone pairs* (p each = 0.02, total p = 0.1) were otherwise similar to the standard pairs, but their intensity was 8 dB lower.

The stimuli in physical and abstract MMN blocks were delivered with an SOA of 510 ms. There were 1300 stimulus pairs in each block, one block lasting 11 min. The presentation order of the different types of stimuli was randomized with the restriction that there always had to be at least one standard stimulus between two deviants.

During the experiment, the participants were sitting on a comfortable reclining chair in an electrically and acoustically shielded room. A computer terminal (diameter 63 cm) for watching the videos and playing Tetris was placed about 130 cm in front of the participant. The participants were instructed to avoid excessive blinking and body movements during the EEG measurements.

There were three different conditions, manipulating the attentional demands of the participants. In the *Video Condition* (VC), the participants were watching a silent movie with subtitles while the auditory stimuli were presented. The participants were instructed to concentrate on watching the movie, ignoring the auditory stimuli presented via the headphones.

In the *Tetris Condition* (TC), the participants were instructed to concentrate on playing the Tetris computer game while the auditory stimuli were presented. The Tetris version used was obtained from the site https://codeincomplete.com/games/tetris/. Although the game was already familiar to all participants, before the experiment they were allowed to practice it for a few minutes, the experimenter ensuring that all the participants understood the idea and the keyboard commands of the game. The participants played the game using the up, down, left, and right buttons of the keyboard with their right-hand forefinger, middle finger, and ring finger for moving and turning the falling shapes. The participants could hold the keyboard either on their lap or on a small table beside the participant. While playing, the participants saw on the screen their score indicating the number of completed lines. They were told that 40 completed lines during each 11-min playing session would be considered a “good result” and they were encouraged to try to get at least that amount of lines. In case the game ended during the auditory stimulation (i.e., the playing area became crowded with the shapes), the participants were instructed to immediately start a new game by pressing the space bar. During the TC, the experimenter could monitor from a parallel terminal in the laboratory the game and ensure that the participant was continuously playing the game as requested.

In the *Detection Condition* (DC), the participants were instructed to concentrate on listening to the auditory stimuli and to press a button with their right-hand index finger always when they noticed a *soft deviant* (either a soft tone or a soft tone pair, depending on the type of the stimulus block). The participants did not have to press the button to pitch deviants or abstract deviants. To avoid the alpha waves in the EEG distorting the ERPs, the participants were instructed to keep their eyes open during the DC. Mean hit rates and reaction times (RTs) to the soft deviants were calculated. A participant's response was classified as a hit if it occurred during 150–650 ms from the onset of the soft deviant. No video or computer game was presented to the participants in the DC.

One physical-MMN and one abstract-MMN block were presented in the VC, TC, and DC, the experiment consisting thus of six stimulus blocks, each block consisting of 1300 tones/tone pairs. The physical and abstract blocks were presented in alternating order. The order of the VC, TC, and DC was counterbalanced across the participants. There was a short break of 1–2 min between each stimulus block, during which the instructions for the next block were given and the participant could have a short rest if needed. With preparations, the experiment lasted about 2 h.

### 2.3 ERP recording and analysis

The EEG (sampling rate 250 Hz, bandpass 1–30 Hz, amplifier: Brainproducts/Brainvision Brainamp DC) was recorded with Ag/AgCl-electrodes placed at Fpz, Fz, F3, F4, Cz, Pz, and the left (LM) and right (RM) mastoids. The electrodes were fixed with SAC2 electrode paste (Spes Medica Srl). The vertical eye movements were recorded with an electrode above the left eye. The reference electrode while recording was attached to the tip of the nose and the grounding electrode was on the forehead.

The EEG was cut to 600-ms epochs starting 100 ms before the onset of the tone (physical MMN blocks) or the first tone of the pair (abstract MMN blocks). The epochs containing EEG changes exceeding ±50 μV were omitted from the averaging. In the abstract-MMN blocks, the ERPs to the various exemplars of the standard pairs were averaged together. The same was done for the different exemplars of the abstract deviants. The mean number of averaged epochs per deviant in the physical-MMN blocks varied between 106 and 115 (depending on the condition) and 61–96 in the abstract-MMN blocks.

The grand-average ERPs were calculated by averaging together the corresponding ERPs from the 15 participants. Difference waves were calculated by subtracting the ERPs to the standard stimuli from those to the deviants. The 100-ms period preceding the onset of the stimulus was used in the difference waves as a baseline except for the abstract deviants, where the 100-ms period preceding the onset of the *second* tone of the tone pair was used as the baseline (as the onset of the second tone was the earliest moment to indicate whether the tone pair was a standard or a reversed pair).

The amplitudes of the various ERP components to the pitch deviants and abstract deviants were calculated from the difference waves as mean amplitudes during specified latency windows, determined on the basis of the grand-average difference waves (see Results). To confirm the presence of the various ERP components, t-tests were used to compare the mean amplitudes to zero. To avoid multiple t-tests, the tests were beforehand concentrated on those electrodes, where the component based on previous literature should be most prominent (MMN: Fz, LM, RM; P3a: Cz; P3b: Pz). Also, the polarity of the component was predetermined to allow the use of directional t-tests (MMN: negative at Fz, positive at LM and RM; P3a: positive; P3b: positive). The possible amplitude differences in the components between VC, TC, and DC were analyzed using repeated-measures ANOVAs.

The primary interest in the present study was in comparing the ERPs to the pitch deviants and direction deviants between VC, TC, and DC. However, the same analyses were performed also for the ERPs to the soft tones as these stimuli were expected to elicit MMNs to intensity changes, thus offering additional data on the possible attention effects on MMN.

## 3 Results

### 3.1 MMNs to pitch deviants and abstract deviants

**Fig. 1:**
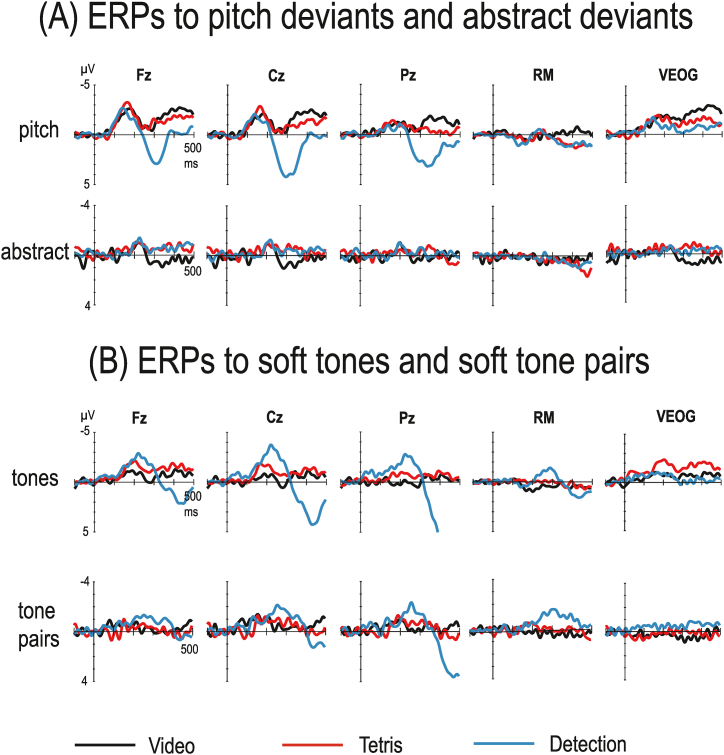
The deviant minus standard difference waves for the pitch deviants and abstract deviants (Panel A), and for the soft tones and soft tone pairs (Panel B) in the video (black lines), Tetris (red), and detection (blue) conditions. Note the differences in μV-scales for various stimuli. The beginning of the tone/tone pair is at the intersection of the x and y axes. For the sake of clarity, mastoid data are shown only from the right mastoid (RM; the ERPs were very similar at both mastoids). VEOG=Vertical electro-oculogram.

Fig. 1A shows the deviant minus standard difference waves for the pitch deviants and abstract deviants (reversed pairs) in VC, TC, and DC. The pitch deviants elicited in all conditions a clear MMN, peaking between 100 and 200 ms. The MMNs were largest at the frontal and central electrodes and reversed polarity at the mastoid electrodes. The MMN was statistically significant in all conditions at Fz (see Table 2). Also, the polarity-inverted MMNs at the mastoid electrodes were mainly statistically significant. In addition to the MMN, a late frontally distributed negativity during 300–500 ms is seen in the VC and TC difference waves. Whether it reflects some later, MMN-related processing remains, however, unclear.

**Table 2:** The mean MMN amplitudes in μV (measurement intervals: pitch deviants 100–200 ms, abstract deviants 200–250 ms) at Fz, LM, and RM in the three conditions. The mean amplitudes were compared to zero with one-tailed t-tests. * = p < 0.05, ** = p < 0.01. The effect sizes (Cohen's d) for the statistically significant MMNs to pitch deviants varied between 0.5 and 1.68 and for the abstract deviants between 0.52 and 0.83.

		Video			Tetris			Detection		
		Fz	LM	RM	Fz	LM	RM	Fz	LM	RM
**pitch deviants**	ampl.	−2.07	0.52	0.32	−2.40	0.72	0.42	−1.94	0.92	0.69
	SD	2.31	0.98	1.34	1.43	1.44	1.28	1.32	0.80	0.89
	t(14)	−3.47**	2.06*	0.95	−6.50**	1.93*	1.26	−5.70**	4.46**	3.01**

**abstract deviants**	ampl.	−0.84	0.31	0.31	−0.97	−0.19	0.22	−1.06	0.25	0.26
	SD	1.61	1.44	1.10	1.55	1.13	1.20	1.27	1.16	0.87
	t(14)	−2.02*	0.84	1.08	−2.43*	−0.64	0.73	−3.22**	0.82	1.14

The abstract deviants elicited in all conditions an MMN, peaking between 200 and 250 ms (i.e., 100–150 ms from the onset of the second tone of the pair, the earliest moment to reveal whether the pair was a standard or a reversed pair). The MMNs were smaller in amplitude than those for the pitch deviants but comparable in size to those typically found in previous abstract-MMN studies. In accordance with many previous abstract-feature MMN studies [10], no clear polarity inversion at the mastoid electrodes was observed. The MMNs were statistically significant in all conditions at Fz (Table 2). In VC, the MMN was followed by a positive deflection. However, this deflection appeared to be caused by an oculomotor artifact of an unknown origin as it was largest at the VEOG electrode. Consequently, it was left unanalyzed.

A two-way 2 × 3 repeated-measures ANOVA (Deviance type: pitch, abstract; Condition: VC, TC, DC) found no statistically significant main effect of Condition for the MMN amplitudes at Fz (F(2,28) = 0.26, p = 0.77, η^2^ = 0.004). The main effect of the Deviance type was statistically significant (F(1,14) = 17.28, p < 0.01, η^2^ = 0.125), indicating that the MMNs to pitch deviants were generally larger in amplitude than those to the abstract deviants. The Deviance type × Condition interaction was not significant (F(2,28) = 0.27, p = 0.76, η^2^ = 0.004).

In DC, the MMN was followed by a large, centrally distributed positive component, peaking at about 300 ms. This component was identified as the P3a (mean amplitude at Cz between 250 and 350 ms 3.77 μV; t(14) = 7.32, p < 0.01).

### 3.2 MMNs to soft tones and soft tone pairs

The difference waves for the soft tones and soft tone pairs are presented in Fig. 1B. The soft tones presented in the physical MMN blocks elicited a prominent negative deflection between 100 and 300 ms. The early part of the deflection (about 100–200 ms) was in all conditions largest at Fz and inverted polarity at the mastoids. Consequently, it was identified as consisting mainly of MMN to an intensity change. The MMN was statistically significant at Fz in all conditions (mean amplitudes during 100–200 ms -0.80 … -1.42 μV; t(14) = -2.10 … -5.15, all p's < 0.05; Cohen's d = 0.54 … 1.33). However, the polarity inverted MMNs at the mastoids were not statistically significant.

In the DC, where the soft tones were the targets, the MMN was followed by the N2b component, as the negativity continued longer than that to pitch deviants and its later part (about 200–350 ms) was largest at Cz and did not invert polarity at the mastoids. The N2b was followed by a very large, parietally distributed P3b component, peaking at about 450 ms.

The MMN to the soft tones at Fz appeared to be slightly larger in TC and DC than in VC. However, a one-way repeated-measures ANOVA found no statistically significant main effect of Condition for the MMN amplitudes at Fz (F(2, 28) = 0.54, p = 0.59, η2 = 0.027).

Unfortunately, for unclear reasons, the ERP data for the soft tone pairs were rather noisy. Some negativity between 100 and 200 ms, indicating a possible MMN, appeared to be present in all conditions but it did not reach statistical significance. In the DC, the MMN was followed by large N2b and P3b components, similar to the soft tones. Due to the lack of statistically significant MMNs, no further analyses for the possible condition effects on MMN were performed.

The mean hit rates for the soft tones and soft tone pairs were 58 % (SD = 21) and 46 % (SD = 20), respectively. The corresponding mean RTs were 475.5 ms (SD = 37.8) and 493.0 ms (SD = 38.4). The mean hit rate was statistically significantly higher for the soft tones than for the soft tone pairs (t(14) = 3.67, p < 0.01). The mean RT was statistically significantly shorter for the soft tones than for the soft tone pairs (t(14) = 2.59, p < 0.05). Both measures indicate that the soft tones were somewhat easier to detect than the soft tone pairs.

## 4 Discussion

The present study was designed to clarify, whether three different types of simple attentional manipulations can influence the MMNs elicited by physical and abstract deviants. The video-watching task, commonly used in current MMN studies, served as a kind of baseline condition. Tetris condition was intended to better control the focusing of attention and prevent attention from wandering in the auditory modality, as the successful playing of the game requires continuous monitoring of and responding to the visual input. In the detection condition, the aim was to find out whether focusing attention on the auditory modality might enhance the MMN. In the present setup the target stimuli, requiring responding, were the soft deviants while the participant did not need to respond to those deviants whose MMN was the actual object of investigation (pitch deviants/abstract deviants). It was hoped that this design could reduce the confounding of MMN with N2b in the ERPs to the latter stimuli. Consequently, the presence of possible attentional effects on the MMN elicited by the pitch deviants and reversed pairs should have been easier to determine.

Both the pitch deviants and abstract deviants elicited a statistically significant MMN in all three conditions. The presence of MMN was confirmed by its frontal midline distribution and the polarity inversion observed at the mastoid electrodes for the pitch deviants. Most importantly, there were no statistically significant MMN amplitude differences between the three conditions to the pitch deviants or reversed pairs. Although the MMN amplitudes were generally larger in the physical MMN blocks than in the abstract MMN blocks, the abstract MMNs were no more susceptible to attention influences than the physical MMNs. These findings add further support for the automaticity of the brain mechanisms underlying the MMN generation.

The smaller MMN amplitudes to abstract deviants compared to those to physical deviants have been observed in several previous studies (see, e.g. Ref. [10]). The present MMN amplitudes and latencies to abstract deviants were close to those observed to similar stimuli in the original Saarinen et al. [11] study (in their Condition 1). Thus our study was also a successful replication of Saarinen et al.‘s main finding. The parameters of the present abstract deviant blocks were almost identical to those of Saarinen et al. However, our SOA was shorter (510 ms vs. 800 ms) and Saarinen et al. ‘s stimulus sequences contained only abstract deviants (no soft tone-pair deviants).

The comparison between VC and TC reveals that the MMN amplitudes were very similar in both conditions. If anything, the MMN amplitudes to physical deviants (pitch deviants and soft tones) tended to be slightly larger in TC than in VC. In case this observation reflects a true but weak effect, it might appear puzzling that the MMN amplitude would *increase* during an attentively more demanding primary task. As both video watching and Tetris tasks relied on visual stimuli, the modality used in the primary task cannot be an explanation. One speculative possibility could be that successful Tetris playing requires continuous alertness whereas during video watching the arousal level of the participant might vary more and there might be periods when it is low (especially if the video turns out to be dull and uninteresting). The amplitude of MMN tends to decrease with decreasing arousal levels (e.g. Ref. [34]).

When the results between VC and DC are compared, it is obvious that the participants performed the detection task in the DC as planned and their attention was indeed directed to the auditory stimuli. This is indicated both by the rather high hit rates for the soft tones and soft pairs, and by the very large N2b and P3b components to these target stimuli. It is also noteworthy that the present setup, where in the DC only the soft stimuli served as the targets, was successful in preventing major N2b contamination on the MMNs to pitch deviants and abstract deviants: there was no indication of N2b to the pitch deviants or abstract deviants (compare the ERPs to pitch deviants/abstract deviants and soft tones/tone pairs in Fig. 1, especially the RM data). As the MMNs to pitch deviants and abstract deviants were very similar both in VC and DC, directing attention to the auditory modality did not seem to essentially increase the MMN amplitude.

The pitch deviants in DC elicited, however, a P3a component, separable from the P3b to the soft tones and soft pairs based on its more central scalp distribution and shorter peak latency. The P3a has been proposed to reflect, among others, mechanisms related to involuntary attention-switching [9]. It can be speculated that as attention in the DC was directed to the auditory modality, the pitch deviants caught the participant's attention, leading to the elicitation of P3a, but as they were not targets, their further processing was discontinued, as indicated by the absence of N2b and P3b components. The reversed pairs did not elicit a similar P3a in DC, perhaps because the abstract deviants were not salient enough to trigger the P3a mechanism.

The MMN data to the soft stimuli provide supplementary evidence for the aforementioned conclusions. The soft tones elicited statistically significant MMNs but again there were no significant amplitude differences between the conditions (when the early part of the difference wave, which also in DC was probably mainly free of N2b contribution, was analyzed). Unfortunately, the ERPs to the soft pairs were rather noisy and the presence of MMN to these stimuli could not be reliably established. The reason for the lack of a clear MMN to soft pairs is unclear. As the soft pairs deviated from the soft tones in the same physical feature (intensity), one should have expected them to produce a large MMN, similarly as did the soft tones. Perhaps the intensity change in two closely succeeding short tones was not as discernible for the MMN system as an intensity change in a single, longer tone.

The results of the present study provide further evidence that the MMN is a robust response to violations in auditory regularities, as no major enhancement or attenuation effects related to present attentional manipulations were observed. We do not claim that no such effects could exist at all, as it is impossible to prove the null hypothesis and there is indeed clear evidence from previous studies (see Introduction) that the MMN is prone to some attentional influences. Due to its exploratory nature, the number of participants in the present study was rather low. However, while the present results do not exclude the possibility that with a larger number of participants, some statistically significant attention effects on MMN could be obtained, their magnitude should be small, having probably little practical implications in most circumstances. Due to the low amplitude of the abstract MMNs, a replication study with a larger amount of participants and better signal-to-noise ratio data would nevertheless be useful to confirm the conclusions concerning the abstract MMNs. Using video watching as a primary task in MMN experiments can be continued as a participant-friendly way of directing attention away from the auditory stimulation. However, in some cases, Tetris (or some other computer game) could be preferred, especially when it is important to better control the possible fluctuations in the participants' attention and vigilance. If needed, the programming code of the game could be modified to enable automatic detection of moments where the participant's attention has probably wandered away from the game, for example, when the participant has temporarily ceased responding by button presses to the forms. EEG epochs related to such phases could then be automatically omitted from the ERP averaging.

## 5 Compliance with ethical standards

Written informed consent was obtained from all participants included in the study. The experimental procedure was carried out in accordance with the Declaration of Helsinki and was ethically accepted by the University of Helsinki Ethical Review Board in Humanities and Social and Behavioral Sciences (approval number: 4/2022).

## CRediT authorship contribution statement

**Petri Paavilainen:** Writing – original draft, Validation, Supervision, Methodology, Formal analysis, Data curation, Conceptualization. **Marianne Ilola:** Writing – original draft, Visualization, Validation, Software, Methodology, Formal analysis, Data curation, Conceptualization.

## Declaration of competing interest

The authors declare that they have no known competing financial interests or personal relationships that could have appeared to influence the work reported in this paper.
